# Living in Historically Redlined Neighborhoods and Biological Aging among Older Adults

**DOI:** 10.1093/gerona/glaf190

**Published:** 2025-10-05

**Authors:** Calley E. Fisk, Katrina M. Walsemann, Chelsey Jones, Eileen Crimmins, Jennifer A. Ailshire

**Affiliations:** a Leonard Davis School of Gerontology, University of Southern California, USA; b University of Maryland, School of Public Policy & Maryland Population Research Center, USA; c Howard University, USA

**Keywords:** Accelerated Aging, Biomarkers, Structural Racism, Neighborhoods, Socioeconomic Status

## Abstract

Living in historically redlined neighborhoods has deleterious effects on aging-related health outcomes, yet little is known about how historical redlining affects the physiological aging process and the role of current neighborhood socioeconomic status (SES) on this relationship. This study determined if living in historically redlined neighborhoods was associated with biological age and if this association was mediated by neighborhood-level socioeconomic status. We linked the Health and Retirement Study 2016 Venous Blood Study (HRS-VBS) to redlining scores from the Historic Redlining Indicator data and census tract level data from the 2014–2018 American Community Survey 5-year estimates (N=6,466 respondents). Multivariable linear regression models were used to assess differences in biological age among older residents of historically redlined neighborhoods graded “Best/Desirable”, “Declining”, and “Hazardous”. Mediation analyses using the khb method were used to assess whether measures of neighborhood affluence and disadvantage explained differences in biological age by historical redlining grade. Older residents of “Declining” or “Hazardous” neighborhoods were about 2.5 and 1.7 years older biologically than residents of “Best/Desirable” neighborhoods. Neighborhood SES mediated this relationship, with affluence explaining approximately 20% and disadvantage explaining about 8% (“Declining”) and 25% (“Hazardous”) of the association between historical redlining and biological age. Our study highlights the importance of evaluating measures of physiological functioning and current neighborhood conditions to clarify existing health disparities among residents of historically redlined neighborhoods.

## Introduction

Historical redlining, a federally sponsored program led by the Home Owner’s Loan Corporation (HOLC) in the 1930s, reinforced and exacerbated the economic and racial segregation of U.S. neighborhoods. By assigning grades to neighborhoods in over 200 cities based largely on area demographics and infrastructure, redlining practices contributed to long-term disparities by systematically limiting economic investment, institutional support, and wealth accumulation in lower-graded, predominantly poor and Black neighborhoods.^1–3^ Recent empirical work finds that current residents of historically lower-graded neighborhoods experience higher mortality^4,5^ and elevated risk for diseases, such as cardiometabolic disorders,^6,7^ certain cancers,^8^ and stroke,^9^ compared to residents of neighborhoods assigned higher redlining grades.

Despite these documented health inequities, few studies have examined the biological mechanisms that likely underlie the relationship between historical redlining and these age-related outcomes. One study among breast cancer patients found a significant association with living in historically redlined areas and age-related changes in DNA methylation.^10^ In contrast, an analysis of Black and Hispanic older adults from the Multi-Ethnic Study of Atherosclerosis found no significant relationship between historical redlining and leukocyte telomere length, one of the hallmarks of aging.^11^ These two studies both focused on specific populations and a narrow set of biological indicators of aging - underscoring the need for research on a broader set of aging biomarkers to better understand the biological impact of historical redlining.

Related research on neighborhood disadvantage – shaped by redlining and other racially discriminatory policies in many U.S. neighborhoods – points to several plausible pathways that may connect historical redlining to multiple biological outcomes. Contemporary neighborhood socioeconomic status conditions have been linked to shorter telomere length,^12^ faster epigenetic age acceleration,^13–15^ higher allostatic load,^16,17^ and increased odds of metabolic syndrome.^18^ Chronic exposure to neighborhood-level stressors may further disrupt biological regulation and accelerated aging at the cellular level.^19,20^ These patterns are especially relevant for the experiences of older adults. Not only does variation in biological functioning increase with age,^21,22^ but older adults are also more vulnerable to their neighborhood environments due to greater reliance on locally available resources, infrastructure, and opportunities for social engagement.^23^

Historical redlining influences contemporary neighborhood socioeconomic status (SES) through lasting effects on local housing markets,^2,3^ homeownership rates,^24^ and property values.^25–27^ Neighborhoods graded as “Declining” or “Hazardous” (grades “C” or “D”) continue to exhibit higher poverty rates,^27^ increased crime,^28,29^ and greater housing instability compared to neighborhoods graded as “Best” or “Still Desirable” (grades “A” or “B”).^30^ These sustained disparities in neighborhood SES can negatively affect biological aging processes in older residents by restricting access to health-promoting resources,^31^ increasing exposure to toxic built environments,^32,33^ and elevating levels of chronic stress.^34^

This study examines whether historical redlining grades are associated with biological age – a multisystem, composite indicator of physiological aging – in older adults. We anticipate that residents of neighborhoods designated as “Declining” or “Hazardous” will exhibit older biological ages than residents of neighborhoods with more favorable historical redlining grades. Additionally, we investigate whether contemporary neighborhood SES mediates the relationship between historical redlining and biological aging.

## Methods

### Data

Individual-level data come from the Health and Retirement Study (HRS), a nationally representative, longitudinal study of U.S. adults over age 50. In 2016 venous blood was collected from 9,934 HRS participants ages 56 and older and assayed for 40 biomarkers across multiple bodily systems (e.g., cardiovascular, immune, inflammation, and renal functioning).^35^ HRS also collects a wide array of sociodemographic and geographic data on respondents. Respondent home addresses were geocoded by HRS and linked to 2010 census tract identifiers, which we used to link respondents to their neighborhood redlining and census data.

Neighborhood data on redlining come from the National Community Reinvestment Coalition (NCRC) Historic Redlining Indicator data (HRI).^36^ The HRI used previously digitized HOLC residential survey data by the Digital Scholarship Lab’s Mapping Inequality Project at the University of Richmond^37^ to aggregate historical redlining grades to the census tract-level. The HRI linked the digitized maps to 2010 census tract boundaries and calculated a historic redlining indicator score representing an area weighted sum of HOLC grades for 12,888 census tracts across the United States. Neighborhood data on census tract-level demographic and economic measures come from the American Community Survey (ACS) 5-year estimates. We use the 2014–2018 ACS 5-year estimates where 2016 represents the mid-year estimates.

### Sample

Our sample includes HRS-VBS respondents who took part in the venous blood collection in 2016 and had a non-missing value on all biomarkers included in our measure of expanded biological age described below (N=6,546). We excluded 80 respondents who were missing data on key variables or covariates, resulting in a final sample size of 6,466 respondents living in 3,396 census tracts.

### Measures

#### Biological Age.

To assess biological age we used the expanded biological age measure previously developed by Crimmins and colleagues^22^ which includes 22 blood-based biomarkers representing multiple systems of biological functioning including cardiovascular (systolic blood pressure, NT-proBNP), metabolic (total cholesterol, HbA1c, IGF 1), and kidney functioning (cystatin C, BUN), as well as markers of inflammation and infection (cytomegalovirus or CMV IgG level, C-reactive protein, albumin, 5 cytokines (IL6, TNFRI, IL-10, IL-1Ra, and TGFB), white blood cell count), immune function (percent lymphocytes, CD4/CD8), hematological markers (mean cell volume, red cell width), and organ function (alkaline phosphatase, expiratory peak flow). Expanded biological age was calculated in two steps. In the first step chronological age was regressed on the biomarkers to obtain the intercept, slope, and mean squared error. In the second step, expanded biological age was estimated by substituting each respondent’s values on the biomarkers using an equation developed in previous aging studies.^21,22^ The final measure of expanded biological age is continuous and, on average, equals the average chronological age for the sample. At the individual level, higher values indicate older biological age, and lower values indicate younger biological age.

Of the original 9,934 HRS-VBS respondents, we exclude respondents from our sample with missing values on any of the 22 biomarkers included in the final biological age measure. Missingness for this variable ranged from <1% (albumin and BUN) to 16.4% (HbA1c). Most biomarkers had <1% missing and the average missingness of all biomarkers was approximately 3.1%. In ancillary models, we imputed biological age to determine if we would find similar results after accounting for missingness on the 22 biomarkers (results described below).

#### Historic Redlining Indicator.

Using the HRI-calculated continuous measure of historical redlining at the census tract-level (range: 1–4), we constructed a categorical indicator of historical redlining. First, we categorized neighborhoods into 4 historical redlining grades using HRI-provided cut points of the continuous measure: “Best” (1.00–1.74), “Desirable” (1.75–2.49), “Declining” (2.50–3.24), and “Hazardous” (3.25–4.00). Next, due to small sample sizes, we combined “Best” and “Desirable” neighborhoods into a single “Best/Desirable” category and included a “No Score” category assigned to HRS-VBS respondents living in neighborhoods without HRI grades. Our final historic redlining indicator variable included the following 4 categories: “Best/Desirable”, “Declining”, “Hazardous”, and “No Score”.

#### Neighborhood Socioeconomic Status.

Two measures of affluence and disadvantage were derived from exploratory factor analysis with orthogonal varimax rotation using the following: percent of the population 25 years or older with 1) less than a high school degree and 2) a bachelor’s degree; percent of the population 3) unemployed, 4) employed in management, professional, and related occupations, 5) below the poverty line, and 6) who own their home; percent of households 7) receiving public assistance; percent of housing units 8) built 30 years ago and 9) vacant; and 10) the median house value and 11) the median household income. Median house value and median household income were adjusted for inflation to match 2021 dollars.

Neighborhood affluence (eigenvalue=4.69) was characterized by high factor loadings on percent with a bachelor’s degree (factor loading=0.94), percent employed in management, professional, and related occupations (0.91) and median household income (0.69). The factor for neighborhood disadvantage (eigenvalue=1.12) was characterized by high factor loadings on percent in poverty (0.68), homeownership (−0.79), and household income (−0.47). Standardized factor scores were generated for all U.S. census tracts prior to merging with the HRS and HRI data. Higher neighborhood affluence scores indicate greater socioeconomic advantage, while higher neighborhood disadvantage scores indicate greater socioeconomic deprivation. Our neighborhood affluence and disadvantage measures are consistent with prior work using factor analytic approaches to measure multidimensional neighborhood socioeconomic status from ACS data.^34^

#### Covariates.

We controlled for respondents’ self-reported race/ethnicity (non-Hispanic White, non-Hispanic Black, non-Hispanic other, and Hispanic), self-reported gender (male and female), educational attainment (measured in years of schooling), and chronological age (measured in years).

### Analytic Approach

We used multivariable linear regression models to predict expanded biological age for respondents in neighborhoods historically rated as Best/Desirable, Declining, Hazardous, or a No Score neighborhood. We estimated a series of 4 models that reflect our conceptualization of the relationships between historical redlining and individual and neighborhood characteristics. Model 1 adjusts for age, sex, and the historical redlining indicator variable. Models 2–3 sequentially add race/ethnicity (Model 2) and years of education (Model 3), both fundamental social determinants of health that influence opportunities across the life course including selection into neighborhoods and wealth accumulation. Model 4 adds tract-level affluence and disadvantage to capture neighborhood-level socioeconomic contexts, which reflect historical redlining practices and disparate community investment as well as independently shape biological aging.

We used the khb command in Stata to decompose the total effect of historical redlining grades in Model 4 into direct and indirect effects on expanded biological age.^38^ Indirect effects of historical redlining grades include the relative contribution of tract-level affluence and disadvantage in explaining our focal relationship. We report the total percentage of the historical redlining coefficients that are explained by the addition of tract-level affluence and disadvantage (i.e., Model 3 to Model 4). Both the mediation analyses and linear regression models were weighted with VBS population weights provided by HRS and estimated using Stata statistical software, version 18. Given that some respondents reside in the same census tract, we report robust standard errors that account for clustering at the tract-level.

## Results

### Sample Characteristics

Table 1 presents descriptive statistics for the study population for the full sample and by historic redlining indicator. The sample had an average biological age of about 69 years (SE=0.24). Most of the sample was White (80.3%), female (54.6%), and had about 13 years of schooling on average (SE=0.06).

Overall, 14% of respondents lived in census tracts with historic redlining indicators (891 respondents living in 549 census tracts). Residents living in neighborhoods historically graded “Declining” (mean=70.94, SE=0.97) and “Hazardous” (mean=69.20, SE=1.43) had slightly older biological ages than those living in “Best/Desirable” neighborhoods (mean=68.89, SE=1.65), despite having slightly younger chronological ages (mean=69.38, SE=0.81 “Declining”; mean=68.12, SE=1.02 “Hazardous”; mean=70.45, SE=1.18 “Best/Desirable).

On average, residents of “Best/Desirable” neighborhoods lived in areas with the highest tract affluence scores and lowest tract disadvantage scores compared to lower-graded areas. Residents of “Declining” and “Hazardous” neighborhoods lived in areas with similar tract affluence scores to one another, while residents of “Declining” neighborhoods lived in areas with lower tract disadvantage scores compared to those in “Hazardous” neighborhoods. Compared to residents of “Best/Desirable” neighborhoods, residents of “Declining” and “Hazardous” neighborhoods were less likely to be White (73.4% vs. 61.3% and 45.9%, respectively) and had fewer years of schooling (mean=13.94, SE=0.32 vs. mean=12.75, SE=0.23 and mean=12.03, SE=0.36, respectively).

Finally, residents of our sample living in “No Score” neighborhoods had an average biological (SE=0.25) and chronological (SE=0.22) age of about 68 years. The biological age of residents of “No Score” neighborhoods was similar to the biological age of the “Best/Desirable” group, while their chronological age was slightly younger. Additionally, residents of “No Score” neighborhoods were more likely to be White (82.9%) and lived in areas with the lowest neighborhood disadvantage score compared to residents of the redlined neighborhoods.

### Regression Results

**Figure 1.**
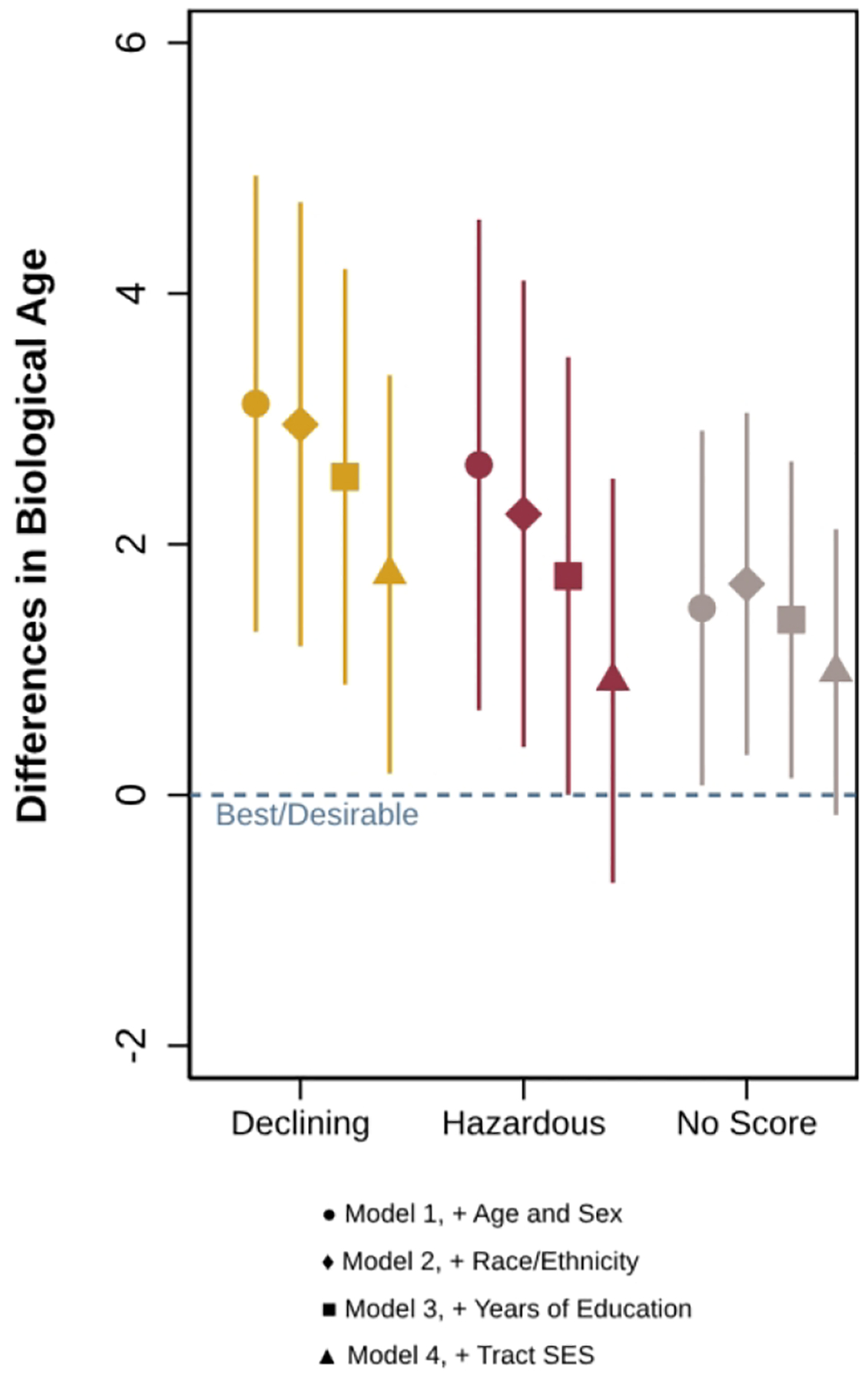
Estimated differences in biological age for residents of “Declining”, “Hazardous”, and “No Score” neighborhoods compared to residents of “Best/Desirable” neighborhoods, Health and Retirement Study Venous Blood Study 2016, Weighted data.

Figure 1 shows results from multivariable linear regression models predicting biological age by historic redlining. A full table with estimates from all models is included in the supplemental material. In Model 1, after adjusting for age and gender, living in neighborhoods with “Declining” and “Hazardous” historic redlining grades was significantly associated with older expanded biological age compared to living in “Best/Desirable” neighborhoods by about 3 years. These results were attenuated but remained significant after including race/ethnicity in Model 2 and adjusting for years of education in Model 3. In Model 3, residents of “Declining” and “Hazardous” neighborhoods were about 2.5 and 1.7 years older biologically than residents of “Best/Desirable” neighborhoods. The inclusion of neighborhood disadvantage and affluence in Model 4 attenuated the relationship between living in “Declining” neighborhoods and expanded biological age compared to “Best/Desirable” neighborhoods (b=1.76, SE=0.81, p<0.05), but the relationship remained statistically significant. The coefficient for living in “Hazardous” neighborhoods (b=0.91, SE=0.82, p=0.27), however, was no longer statistically significant in Model 4.

Decomposition estimates are presented in Table 2 and indicate that tract affluence explained approximately 22% of the difference in biological age between residents of “Declining” and “Best/Desirable” neighborhoods and between residents of “Hazardous” and “Best/Desirable” neighborhoods. Tract disadvantage explained about 9% of the difference in biological age between residents of “Declining” and “Best/Desirable” neighborhoods and about 25% of the difference between residents of “Hazardous” and “Best/Desirable” neighborhoods. In models where tract affluence and tract disadvantage were included one at a time, both tract affluence and disadvantage independently reduced the “Hazardous” coefficient to statistical non-significance (results available upon request).

### Ancillary Results

We ran a series of ancillary analyses to support our main findings. Results for all ancillary analyses are available in supplementary materials. First, due to missingness on our biological age variable, we re-estimated our linear regression models using an imputed measure of biological age. Imputation models were conducted using the mi *impute* command with chained equations in Stata, version 18 and estimated separately for men and women. Results were similar to the main analysis, although overall effect sizes were slightly attenuated (Supplemental Table A2). In Model 4, residents of “Declining” neighborhoods were about 1.4 years (SE=0.66, p=0.03) older biologically than residents of “Best/Desirable” neighborhoods when predicting imputed biological age and adjusting for individual sociodemographics and neighborhood SES. For the mediation analysis, tract affluence explained about 26% of the difference in biological age between residents of “Declining” and “Best/Desirable” neighborhoods and about 34% of the difference between residents of “Hazardous” and “Best/Desirable” neighborhoods. Decomposition estimates for tract disadvantage were comparable to the main analysis for all redlined neighborhoods.

Second, to evaluate whether specific biological systems disproportionally drive our observed association between historical redlining and biological age, we estimated separate models for inflammation (a composite measure of C-reactive protein, albumin, percent lymphocytes, and 5 cytokines (IL6, TNFRI, IL-10, IL-1Ra, and TGFB)), renal functioning (a composite measure of creatine and cystatin C), and a metabolic risk score (a count of clinically defined high risk scores on total cholesterol, HbA1c, systolic blood pressure, and waist circumference) (Supplemental Table A3a). We also estimated separate models for measures of immune functioning (cytomegalovirus seropositivity and presence of CD4 and CD8 T cells) (Supplemental Table A3b). Except for inflammation, we found no significant association between historical redlining and these outcomes or no evidence for a mediation effect of neighborhood SES on this relationship. For inflammation, effect sizes for residents of “Declining” neighborhood were significant but small, suggesting that there may not be a single system responsible for differences in biological age among residents of redlined neighborhoods.

Finally, given that historical redlining practices contributed to unequal living environments for individuals racialized as Black and White, we stratified our models by race/ethnicity (non-Hispanic White (N=4,405), non-Hispanic Black (N=1,012), and Hispanic (N=864); Supplemental Table A4). Findings for White older adults are similar to our main findings. For Black adults, effect sizes for residents of “Declining” neighborhoods were much smaller than in the overall sample, whereas effect sizes for residents of “Hazardous” neighborhoods were larger. Among Hispanic older adults, effect sizes were attenuated, yet the association between historical redlining and biological age was consistent with the main analysis. However, findings for both Black and Hispanic older adults were not statistically significant, and our race-stratified analyses are likely underpowered due to small sample sizes of those in redlined neighborhoods, particularly among our Hispanic sample.

## Discussion

Neighborhood conditions linked to biological aging reflect sociohistorical processes that continue to shape contemporary inequalities in living environments and systemic disenfranchisement. By integrating neighborhood-level historical data with individual-level biological markers, this study provides evidence that historical redlining – a structural manifestation of such sociohistorical processes – is related to accelerated aging among older residents. We show that residents living in historically lower-graded neighborhoods exhibited accelerated biological aging by 1.5–2.5 years compared to residents of neighborhoods historically assigned higher grades, independent of individual-level characteristics.

These findings are consistent with recent literature on historical redlining and aging-related health outcomes. Studies have shown that residents of lower-graded historically redlined neighborhoods have worse cardiovascular health and higher mortality rates than residents of higher-graded neighborhoods.^39–41^ The differences between graded neighborhoods can be large; one study found a 5-year difference in life expectancy at birth between the best and worst-graded neighborhoods.^42^ Although we found slightly smaller differences when investigating biological age (1–2 years), these patterns likely capture health risks associated with common conditions among older adults as biological age is a known predictor of chronic disease and mortality.^21,22^ Our results also highlight the physiological consequences of place-based inequities and the potential biological mechanisms underlying the redlining-health relationship, suggesting that 1) health differences between residents of redlined neighborhoods are present at the biological level and may precede the development of observed conditions and 2) the increased multisystem dysregulation experienced by residents of lower-graded neighborhoods may be one explanatory pathway connecting residence in lower-graded neighborhoods to poorer health outcomes.

We also found that the inclusion of neighborhood affluence and disadvantage explained differences in biological aging among those in “Declining” neighborhoods compared to “Best/Desirable” neighborhoods, and reduced differences in biological age to non-significance for residents of “Hazardous” neighborhoods. A review of the literature found only a few studies that have explicitly examined the mediating effect of neighborhood socioeconomic conditions on the relationships between historically redlining and health.^43^ These studies have found that variation in neighborhood property values, social vulnerability scores, and socioeconomic status account for disparities in life expectancy, receipt of cancer treatment, and reports of poor physical and mental health among residents of historically-redlined neighborhoods.^39,44,45^

To our knowledge, this is the first study to examine how both advantaged and disadvantaged neighborhood socioeconomic conditions link historical redlining to biological aging. For “Declining” neighborhoods, neighborhood affluence accounted for more of this association than neighborhood disadvantage. Residents in higher-graded, more affluent neighborhoods may benefit from greater access to health-promoting resources, such as quality health care and reduced exposure to chronic stress.^46^ Some of the biological aging differences between “Declining” and “Best/Desirable” neighborhoods may also stem from unmeasured aspects of neighborhood affluence. For example, wealthier, higher-graded neighborhoods have fewer dilapidated buildings and more greenspace than poorer, lower-graded neighborhoods.^47^ These neighborhood factors can reduce residents’ exposure to environmental stressors like air pollution and heat – both of which are linked to biological aging.^20,48,49^

Conversely, for “Hazardous” neighborhoods, neighborhood disadvantage accounted for a greater share of the association between historical redlining and biological age compared to neighborhood affluence. Current neighborhood disadvantage likely reflects both historical inequities established at the time of redlining and sustained disinvestment, which limited the development and preservation of health-promoting social and economic resources present in “Best/Desirable” neighborhoods. Residents in these disadvantaged environments may experience chronic, socioeconomic stressors, leading to heightened physiological responses and accelerated biological aging. Indeed, higher levels of perceived and observed neighborhood stress and lower social support are related to higher levels of cortisol and faster telomere attrition.^11,50,51^

Our results should be considered given study limitations. First, we include neighborhoods not covered by historical redlining maps to capture the full range of neighborhoods represented in the national HRS-VBS sample. However, we cannot reliably interpret the results for “No Score” neighborhoods, as this category includes both areas that developed after the redlining era and redlined neighborhoods that were not captured in the HRI data. Additionally, the crosssectional design – limited to the HRS 2016 sample and the 2014–2018 neighborhood estimates – does not assess long-term exposure to neighborhood conditions and we do not know how long HRS respondents may have lived in a redlined neighborhood prior to their inclusion in the HRS sample. It is possible that longer-term residents of lower-graded neighborhoods may experience more accelerated aging due to prolonged exposure to disadvantaged environments. Using the geocoded census tract identifiers for the entire HRS observation period (1992–2018), we calculated the length of current neighborhood residence in 2016. However, in ancillary analysis testing whether length of residence moderated the redlining-biological age association, we found no significant differences between shorter- and longer-term residents (results available upon request).

Finally, our analysis does not include other potential mediators that may be influenced by the effects of historical redlining on current neighborhood socioeconomic status, such as health behaviors. In supplemental analysis, we estimated an additional model that included four health-related measures - alcohol consumption, smoking status, physical activity, and BMI - and found results consistent with those presented in Model 4. After adjusting for these health measures, residents of “Declining” neighborhoods were about 1.5 years older biologically (SE=0.75, p<0.05) than residents of “Best/Desirable” neighborhoods (results available upon request). However, because biological aging is measured cross-sectionally and our models lack full life course data on residential history and health behaviors, the temporal ordering of these relationships remains uncertain. Future research using longitudinal data and repeated measures of biological aging is needed to more fully assess the indirect pathways linking historical redlining to biological aging via neighborhood conditions, health, and health behaviors.

### Conclusions

Our findings contribute to understanding health disparities among adults living in historically redlined neighborhoods by examining the links between historical redlining, neighborhood SES, and biological aging. We extend prior research on neighborhood SES and physiological aging by emphasizing the role of sociohistorical forces in shaping contemporary neighborhood conditions. We also investigate these relationships among older adults, who may be especially vulnerable to the health impact of historical redlining due to accumulated physiological wear and greater reliance on neighborhood resources. Using neighborhood-level redlining data and individual-level biomarker data, we found that residents of lower-graded neighborhoods were biologically 2 to 3 years older, on average, than those in higher-graded neighborhoods, after adjusting for individual demographics. Neighborhood affluence and disadvantage explained 1 to 2 years of the overall difference, but, even after accounting for these factors, residents of “Declining” neighborhoods were biologically about 1.5 years older than those in “Best/Desirable” neighborhoods.

Our results highlight how historical redlining remains linked to present-day neighborhood conditions that shape biological vulnerability in older adults. Future research that integrates measures of structural racism can deepen our understanding of how neighborhood environments become biologically embedded over time and inform efforts to reduce health disparities and promote healthy aging.

## Supplementary Material

jgbs_supplemental_fisk2025

## Figures and Tables

**Table 1.** Sample Characteristics and Bivariate Associations by Historic Redlining Indicator, HRS 2016, N=6,466, weighted analysis.

	Total Mean(SE) or %	Historic Redlining Indicator			
		Best/Desirable Mean(SE) or %	Declining Mean(SE) or %	Hazardous Mean(SE) or %	No Score Mean(SE) or %
Expanded Biological Age (46.8–117.8)	69.02 (0.24)	68.89 (1.65)	70.94 (0.97)	69.20 (1.43)	68.91 (0.25)^b^
Biological Age Acceleration (−18.1–43.7)	0.00 (0.12)	−1.56 (0.71)	1.56 (0.60)^a^	1.07 (0.69)^a^	−0.07 (0.12)^ab^
Age (57–100)	69.02 (0.21)	70.45 (1.18)	69.38 (0.81)	68.12 (1.02)	68.98 (0.22)
Race/Ethnicity					
White, non-Hispanic	80.3	73.4	61.3	45.9^ab^	82.9^abc^
Black, non-Hispanic	8.6	19.4	24.4	27.9	6.6^abc^
Hispanic	8.5	5.1	10.4^a^	23.5^ab^	7.9^c^
Other, non-Hispanic	2.6	2.1	3.9	2.7	2.6
Gender					
Male	45.4	44.2	45.9	40.3	45.6
Female	54.6	55.8	54.1	59.7	54.4
Years of schooling (0–17)	13.37 (0.06)	13.94 (0.32)	12.75 (0.23)^a^	12.03 (0.36)^a^	13.43 (0.06)^bc^
Tract-level Socioeconomic Status					
Affluence (z-score: −1.8–3.1)	0.01 (0.02)	0.66 (0.18)	−0.14 (0.09)^a^	−0.07 (0.14)^a^	0.00 (0.02)^a^
Disadvantage (z-score: −1.9–4.2)	−0.18 (0.02)	0.07 (0.12)	0.60 (0.09)^a^	1.16 (0.13)^ab^	−0.28 (0.02)^abc^
Respondents	6,466	238	366	287	5,575
Tracts	3,396	146	224	179	2,847

Notes: Biological age acceleration measured using residuals from linear regression models predicting expanded biological age net of chronological age.

a significantly different from Best/Desirable at p<0.05 level, two-tailed test

b significantly different from Declining at p<0.05 level, two-tailed test

c significantly different from Hazardous at p<0.05 level, two-tailed test

**Table 2.** Linear Regression Models and Decomposition Estimates predicting Biological Age by Historic Redlining Indicator, HRS 2016, N=6,466, weighted analyses.

	Model 3	Model 4	*Percent of HRI coefficient explained by*	
	b(SE)	b(SE)	Tract Affluence	Tract Disadvantage
Historic Redlining Indicator (HRI)				
Best/Desirable (REF)				
Declining	2.54 (0.85)**	1.76 (0.81)*	21.9%***	8.8%*
Hazardous	1.75 (0.89)*	0.91 (0.82)	22.5%*	25.4%**
No Score	1.40 (0.64)*	0.98 (0.58)	40.3%***	−10.5%*
Tract-level Socioeconomic Status				
Affluence		−0.86 (0.15)***		
Disadvantage		0.50 (0.16)**		
Constant	73.61 (0.87)***	73.01 (0.83)***		

Notes: Robust standard errors reported to account for multiple respondents in census tracts. HRI = Historic Redlining Indicator, REF = reference. All models adjust for age, gender, race/ethnicity, and years of education. Decomposition estimates for percent of HRI coefficient explained by tract-level socioeconomic status derived from the khb method.^38^

*** p<0.001,

** p<0.01,

* p<0.05
