# Supplementary material for: Living in Historically Redlined Neighborhoods and Biological Aging among Older Adults
Source: J Gerontol A Biol Sci Med Sci. Author manuscript; Available in PMC 2025 Dec 8. (PMC12510732; doi:10.1093/gerona/glaf190)
Supplement: jgbs_supplemental_fisk2025 [file NIHMS2115538-supplement-jgbs_supplemental_fisk2025.pdf]

Supplemental Table A1. Linear Regression Models predicting Biological Age by Historic Redlining Indicator, HRS 2016, N=6,466, weighted analyses.

	Model 1	Model 2	Model 3	Model 4
	b(SE)	b(SE)	b(SE)	b(SE)
Historic Redlining Indicator				
Best/Desirable (REF)				
Declining	3.12 (0.93)***	2.96 (0.90)**	2.54 (0.85)**	1.76 (0.81)*
Hazardous	2.63 (1.00)**	2.24 (0.95)*	1.75 (0.89)*	0.91 (0.82)
No Score	1.49 (0.72)*	1.68 (0.70)*	1.40 (0.64)*	0.98 (0.58)
Age (divided by 10)	10.01 (0.13)***	10.04 (0.13)***	9.83 (0.13)***	9.81 (0.13)***
Female (REF=Male)	-0.02 (0.21)	-0.07 (0.21)	-0.21 (0.20)	-0.20 (0.20)
Race/Ethnicity				
White, non-Hispanic (REF)				
Black, non-Hispanic		1.78 (0.41)***	1.16 (0.41)**	0.49 (0.42)
Hispanic		1.35 (0.36)***	-0.51 (0.42)	-1.05 (0.41)*
Other, non-Hispanic		0.45 (0.58)	0.29 (0.56)	0.06 (0.57)
Years of Education			-0.44 (0.04)***	-0.35 (0.04)***
Tract-level Socioeconomic Status				
Affluence				-0.86 (0.15)***
Disadvantage				0.50 (0.16)**
Constant	67.54 (0.72)***	67.14 (0.70)***	73.61 (0.87)***	73.01 (0.83)***

Notes: Age mean centered at 69 years old, Robust standard errors reported to account for multiple respondents in census tracts. REF = reference

***p<0.001, **p<0.01, *p<0.05

Supplemental Table A2. Linear Regression Models and Decomposition Estimates predicting Imputed Biological Age by Historic Redlining Indicator, HRS 2016, N=8,707, weighted analyses.

	Model 1	Model 2	Model 3	Model 4	*Percent of HRI coefficient explained by*	
	b(SE)	b(SE)	b(SE)	b(SE)	Tract Affluence	Tract Disadvantage
Historic Redlining Indicator (HRI)						
Best/Desirable (REF)						
Declining	2.88 (0.75)***	2.63 (0.72)***	2.20 (0.68)**	1.43 (0.66)*	26.4%***	8.7%*
Hazardous	2.36 (0.79)**	1.88 (0.75)*	1.35 (0.71)	0.56 (0.67)	34.2%**	24.5%**
No Score	1.31 (0.59)*	1.51 (0.56)**	1.15 (0.52)*	0.68 (0.48)	51.1%***	-10.7%*
Age (divided by 10)	10.02 (0.10)***	10.07 (0.10)***	9.84 (0.10)***	9.84 (0.10)***		
Female (REF=Male)	-0.02 (0.18)	-0.06 (0.18)	-0.20 (0.18)	-0.21 (0.18)		
Race/Ethnicity						
White, non-Hispanic (REF)						
Black, non-Hispanic		2.01 (0.36)***	1.42 (0.36)***	0.88 (0.37)*		
Hispanic		1.45 (0.29)***	-0.43 (0.33)	-0.87 (0.33)**		
Other, non-Hispanic		0.37 (0.45)	0.14 (0.42)	-0.01 (0.42)		
Years of Education			-0.43 (0.03)***	-0.35 (0.04)***		
Tract-level Socioeconomic Status						
Affluence				-0.78 (0.12)***		
Disadvantage				0.40 (0.14)**		
Constant	67.65 (0.59)***	67.18 (0.57)***	73.61 (0.71)***	73.13 (0.69)***		

Notes: Age mean centered at 69 years old, Robust standard errors reported to account for multiple respondents in census tracts. HRI = Historic Redlining Indicator, REF = reference. Decomposition estimates for percent of HRI coefficient explained by tract-level socioeconomic status derived from the khb method.

***p<0.001, **p<0.01, *p<0.05

Supplemental Table A3a. Linear Regression Models and Decomposition Estimates predicting Biological System Functioning (Inflammation, Renal Functioning, and Metabolic Risk) by Historic Redlining Indicator, HRS 2016, weighted analyses.

			Percent of HRI coefficient explained by	
	Model 3 b(SE)	Model 4 b(SE)	Tract Affluence	Tract Disadvantage
Inflammation (N=6,465)				
Historic Redlining Indicator (HRI)				
Best/Desirable (REF)				
Declining	0.18 (0.08)*	0.11 (0.08)	30.7%***	6.2%
Hazardous	0.08 (0.11)	0.02 (0.10)	48.5%*	27.7%
No Score	0.05 (0.07)	-0.00 (0.07)	123.9%***	-16.3%
Tract-level Socioeconomic Status				
Affluence		-0.09 (0.01)***		
Disadvantage		0.03 (0.02)		
Constant	0.20 (0.09)*	0.14 (0.09)		
Renal Functioning (N=6,465)				
Historic Redlining Indicator (HRI)				
Best/Desirable (REF)				
Declining	0.11 (0.07)	0.07 (0.07)	28.8%**	3.3%
Hazardous	0.04 (0.06)	0.01 (0.07)	62.0%*	19.8%
No Score	0.07 (0.04)	0.05 (0.05)	41.9%**	-3.1%
Tract-level Socioeconomic Status				
Affluence		-0.05 (0.01)***		
Disadvantage		0.01 (0.02)		
Constant	0.87 (0.07)***	0.84 (0.07)***		
Metabolic Risk Score (N=5,059)				
Historic Redlining Indicator (HRI)				
Best/Desirable (REF)				
Declining	0.15 (0.13)	0.02 (0.14)	81.8%***	6.1%
Hazardous	0.31 (0.13)*	0.21 (0.14)	26.0%	5.6%
No Score	0.13 (0.10)	0.01 (0.12)	96.6%***	-5.1%
Tract-level Socioeconomic Status				
Affluence		-0.18 (0.03)***		
Disadvantage		0.02 (0.03)		
Constant	2.95 (0.15)***	2.84 (0.16)***		

Notes: Robust standard errors reported to account for multiple respondents in census tracts. HRI = Historic Redlining Indicator, REF = reference. All models adjust for age, gender, race/ethnicity, and years of education. Decomposition estimates for percent of HRI coefficient explained by tract-level socioeconomic status derived from the khb method.

***p<0.001, **p<0.01, *p<0.05

Supplemental Table A3b. Linear (Cd4 and CD8) and Logistic (CMV) Regression Models and Decomposition Estimates predicting Immune Functioning by Historic Redlining Indicator, HRS 2016, weighted analyses.

			Percent of HRI coefficient explained by	
	Model 3 b(SE)	Model 4 b(SE)	Tract Affluence	Tract Disadvantage
CD4 T Cells (N=6,465)				
Historic Redlining Indicator (HRI)				
Best/Desirable (REF)				
Declining	0.05 (0.05)	0.04 (0.05)	14.6%	0.6%
Hazardous	-0.02 (0.05)	-0.02 (0.05)	-31.3%	-3.7%
No Score	0.05 (0.03)	0.04 (0.03)	16.1%	-0.5%
Tract-level Socioeconomic Status				
Affluence		-0.01 (0.01)		
Disadvantage		0.00 (0.01)		
Constant	0.85 (0.05)***	0.84 (0.05)***		
CD8 T Cells (N=6,465)				
Historic Redlining Indicator (HRI)				
Best/Desirable (REF)				
Declining	0.05 (0.03)	0.04 (0.03)	23.4%**	3.0%
Hazardous	0.05 (0.03)	0.04 (0.03)	15.7%*	5.7%
No Score	0.01 (0.02)	-0.00 (0.02)	129.4%**	-10.8%
Tract-level Socioeconomic Status				
Affluence		-0.02 (0.00)***		
Disadvantage		0.00 (0.01)		
Constant	0.39 (0.03)***	0.38 (0.03)***		

			Percent of HRI coefficient explained by	
	Model 3 OR(95% CI)	Model 4 OR(95% CI)	Tract Affluence	Tract Disadvantage
CMV Seropositivity Status (N=6,465)				
Historic Redlining Indicator (HRI)				
Best/Desirable (REF)				
Declining	1.27 (0.74-2.17)	1.01 (0.59-1.72)	64.6%**	30.8%*
Hazardous	1.74 (0.95-3.19)	1.36 (0.74-2.47)	19.3%*	25.9%*
No Score	1.36 (0.89-2.09)	1.22 (0.80-1.86)	50.2%**	-15.5%*
Tract-level Socioeconomic Status				
Affluence		0.79 (0.72-0.87)***		
Disadvantage		1.18 (1.05-1.31)**		
Constant	3.54 (1.93-6.49)***	2.90 (1.59-5.28)***		

Notes: Robust standard errors reported to account for multiple respondents in census tracts. HRI = Historic Redlining Indicator, REF = reference. All models adjust for age, gender, race/ethnicity, and years of education. Decomposition estimates for percent of HRI coefficient explained by tract-level socioeconomic status derived from the khb method.

***p<0.001, **p<0.01, *p<0.05

Supplemental Table A4. Linear Regression Models and Decomposition Estimates predicting Biological Age by Race/Ethnicity and Historic Redlining Indicator, HRS 2016, weighted analyses.

			Percent of HRI coefficient explained by	
	Model 3<br>b(SE)	Model 4<br>b(SE)	Tract<br>Affluence	Tract<br>Disadvantage
White, Non-Hispanic (N=4,405)				
Historic Redlining Indicator (HRI)				
Best/Desirable (REF)				
Declining	3.03 (1.11)**	2.19 (1.07)*	18.6%**	9.2%*
Hazardous	1.76 (0.95)	0.72 (0.90)	25.2%	34.0%**
No Score	1.77 (0.75)*	1.37 (0.68)*	34.0%**	-11.5%
Tract-level Socioeconomic Status				
Affluence		-0.79 (0.17)***		
Disadvantage		0.66 (0.20)***		
Constant	74.94 (1.05)***	74.34 (1.02)***		
Black, Non-Hispanic (N=1,012)				
Historic Redlining Indicator (HRI)				
Best/Desirable (REF)				
Declining	0.43 (1.39)	0.30 (1.41)	31.2%	-0.4%
Hazardous	1.96 (2.13)	1.84 (2.01)	6.4%	-0.2%
No Score	-0.03 (1.23)	-0.05 (1.26)	-64.0%	-4.0%
Tract-level Socioeconomic Status				
Affluence		-0.63 (0.44)		
Disadvantage		-0.00 (0.45)		
Constant	74.18 (2.00)***	73.48 (2.14)***		
Hispanic (N=864)				
Historic Redlining Indicator (HRI)				
Best/Desirable (REF)				
Declining	2.07 (1.32)	0.91 (1.44)	44.7%*	11.5%
Hazardous	0.62 (1.15)	0.05 (1.21)	31.9%	60.0%
No Score	0.90 (0.84)	0.08 (0.92)	102.7%*	-11.4%
Tract-level Socioeconomic Status				
Affluence		-1.45 (0.35)***		
Disadvantage		0.43 (0.34)		
Constant	71.52 (1.33)***	70.24 (1.43)***		

Notes: Robust standard errors reported to account for multiple respondents in census tracts. HRI = Historic Redlining Indicator, REF = reference. All models adjust for age, gender, race/ethnicity, and years of education. Decomposition estimates for percent of HRI coefficient explained by tract-level socioeconomic status derived from the khb method.

***p<0.001, **p<0.01, *p<0.05
